# A comparison of the sensory and rheological properties of molecular and particulate forms of xanthan gum^☆^

**DOI:** 10.1016/j.foodhyd.2013.04.018

**Published:** 2014-03

**Authors:** Rachael Abson, Sanyasi R. Gaddipati, Joanne Hort, John R. Mitchell, Bettina Wolf, Sandra E. Hill

**Affiliations:** a Division of Food Sciences, Sutton Bonington Campus, University of Nottingham, Leicestershire LE12 5RD, UK; b Biopolymer Solutions Ltd, Sutton Bonington Campus, University of Nottingham, Leicestershire LE12 5RD, UK

**Keywords:** Xanthan, Extrusion, Flavour perception, Starch, Rheology, Particulate structure

## Abstract

A particulate form of xanthan gum was prepared by extrusion cooking. The temperature dependence of the viscosity of this form shows similarities to starch with an increase in viscosity to a maximum with increasing temperature as a result of the swelling of the particles. The rheology and mixing behaviour with water of the particulate and conventional molecular forms of xanthan were compared with a modified starch. The particulate xanthan products mixed rapidly with water in a similar way to ungelatinised starch, whereas conventional molecular xanthan systems mixed poorly. Using an experienced sensory panel, model tomato products thickened with the three systems were compared at equal shear viscosities. The panel could not discriminate between the tomato flavour of the three products, but found that the xanthan products were perceived as being significantly thicker. These observations were consistent with previous work. Salt perception for both xanthan products was poorer than for the starch thickened systems. A hypothesis to explain why xanthan does not fit into the previously postulated link between mixing and perception is presented.

## Highlights

• Particulate form of xanthan was made by extrusion, no degradation of biopolymer.
• Suspension of the particulate form of xanthan mixed well with water.
• No difference in flavour of soups thickened by starch or two forms of xanthan.
• No link between the mixing behaviour and flavour perception for xanthan.
• Suggested that xanthan interacts in a specific way with saliva.

## Graphical abstract

**Figure:**
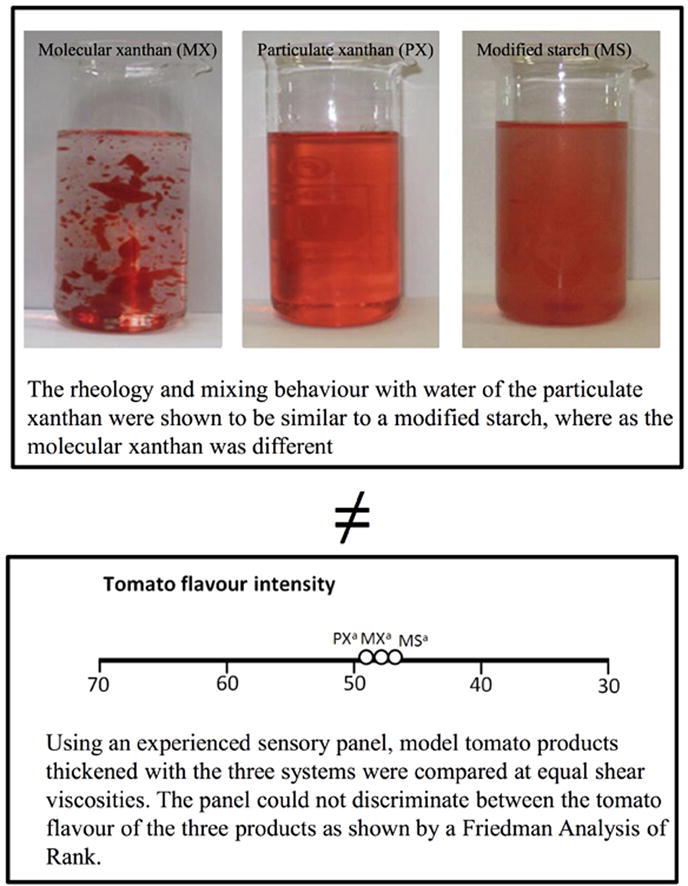

## 1 Introduction

It is well recognised when the viscosity of a hydrocolloid thickened product is increased there is a decrease in flavour and taste perception. This generally occurs once the hydrocolloid concentration exceeds the c* concentration (Baines and Morris, 1987; Cook, Hollowood, Linforth & Taylor, 2003; Hollowood, Linforth & Taylor, 2002; Koliandris, Lee, Ferry, Hill & Mitchell, 2008; Morris, 1993). At the c* concentration, sometimes called the overlap concentration, the hydrodynamic volumes of the polysaccharide coils start to closely pack in the solution and the rheological properties of the solution changes from dilute to semi-dilute. A detailed definition and discussion of this concentration is given by Lapasin and Pricl (1999). However, analysis of nose space volatiles during consumption showed no decrease in intensity when the c* concentration is exceeded (Hollowood et al., 2002) so it seems unlikely that this decrease in taste and flavour is due to a reduction in the amount of volatiles reaching the sensing organs. Cook et al. (2003) showed that an increase in viscosity as measured by the Kokini oral shear stress or other viscosity related parameters could be directly related to taste perception. However, there is evidence that high viscosity products thickened with starches show good taste and flavour perception in contrast to products thickened with most hydrocolloids (Ferry et al., 2006; Hill, Mitchell & Sherman, 1995). Thus different perception magnitudes can be obtained at the same value of viscosity parameters such as the Kokini oral shear stress.

It was suggested by Baines and Morris (1987) that restricted mixing between the hydrocolloid solution and saliva could be the reason for the reduction in taste and flavour perception above c*. To test this hypothesis Ferry and co-workers (Ferry et al., 2006) visualised mixing by including a small amount of red dye in the hydrocolloid solution and gently mixing the solution by hand with water. This showed clearly that at high viscosities starch thickened products generally mixed much more readily with water and presumably saliva than products thickened with hydrocolloids above the c* concentration. For starch the efficiency of mixing was less good if the granule structure had been disrupted as would be the case when unmodified waxy maize starch was subjected to shear following gelatinisation or as a result of the action of amylase. Thus even though there will be a reduction in viscosity within the mouth as a result of amylase activity within the time scale of consumption (Ferry, Hort, Mitchell, Lagarrigue & Pamies, 2004) because the enzyme disrupts the granular structure of starch, subjects with a higher amylase activity perceive taste less well (Ferry et al., 2006). The differences in the ease of mixing of hydrocolloid solutions and suspensions of swollen starch granules can be understood from the ease of breakup of viscous liquid droplets. Droplets thickened by granular starch broke up more readily than droplets thickened by hydrocolloid solutions (Desse, Mitchell, Wolf & Budtova, 2011).

From the above arguments it follows that if the structure of a hydrocolloid solution could be converted from a molecular solution to a particulate suspension resembling starch, then taste and flavour perception at high viscosities should be improved. We have previously demonstrated that by extrusion processing xanthan gum can be converted to a particulate form (Sereno, Hill & Mitchell, 2007). In this previous paper it was suggested that the xanthan structure was reorganised during the extrusion process. A consequence of this reorganisation is the creation of polyelectrolyte particles maintained by xanthan dihelical junction. An investigation aimed at understanding why this occurs in the extrusion process but not during conventional heating is being undertaken and will be the subject of a separate submission.

In the current paper we compare the properties of particulate and molecular (conventional) xanthan and a modified starch in terms of rheology, mixing behaviour and sensory perception. The main objective of the work was to test the hypothesis that the particulate form of xanthan would show better taste and flavour perception than the conventional form at comparable viscosities.

## 2 Materials

Xanthan gum (Keltrol-T) was a gift from CP Kelco and a modified waxy maize starch (ColFlo 67) came from National Starch. Tomato juice with a declared salt content of 1.7 mg NaCl/100 ml was purchased from a local supermarket. Deionised water was used for the rheological and viscosity measurements on the aqueous systems, but tap water was used for the soups tested by the panellists.

## 3 Methods

### 3.1 Preparation of extruded xanthan

The xanthan powders were processed using a Prism TSE 24 MC (Thermo Fisher, UK) twin screw extruder. The powder and water feed rates were 2 kg/h and 1.4 l/h and the screw speed was 300 rpm.

**Fig. 1:**
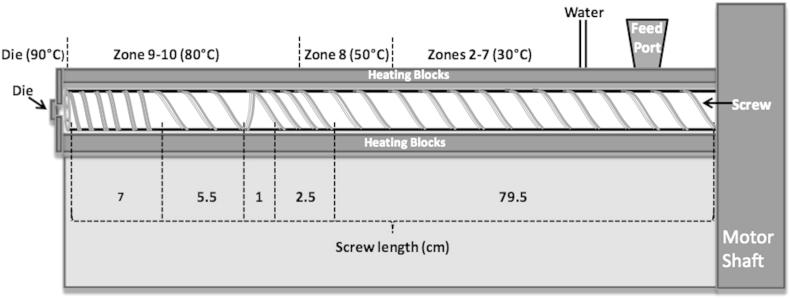
Screw configuration and barrel temperature profiles of the extruder.

The heater temperatures and screw profiles are shown in Fig. 1.

A ribbon die with a slit dimension of 30 × 1 mm was used. Pressure and temperature behind the die was determined. Residence time was measured by including a small amount of red dye in the water feed and measuring the time for the change in colour of the product exiting the die to be observed. After exiting the die the product was dried at a temperature of 70 °C in a conventional oven. The dried material was milled using a Perten laboratory mill 3600 (Perten, UK) and sieved to give samples in the particle size range of 250–425 microns.

### 3.2 Rheological characterisation of xanthans, starch and soups

#### 3.2.1 Rapid viscosity analysis

Information about the change in viscosity with temperature was obtained using a Rapid Viscosity Analyser (Newport Scientific, Australia) operating under conditions frequently used to monitor starch pasting. The appropriate weight of starch or xanthan powder was added to 30 g of a 0.2% solution of NaCl in water maintained initially at a temperature of 25 °C. After holding at this temperature for 2 min the sample was heated at 14 °C/min to 95 °C, held at this temperature for 5 min and cooled to 25 °C. The sample was continuously stirred at 160 rpm.

#### 3.2.2 Intrinsic viscosity

To determine if the extrusion process had degraded the xanthan the zero shear intrinsic viscosity was measured in 0.2% NaCl at 20 °C. An Anton Paar MCR 301 rheometer equipped with concentric cylinder geometry was used. Solutions of extruded xanthan were taken from the RVA canister after the sample had gone through the full temperature profile described above. Measurements were also made on solutions of the original non-processed material. The samples were diluted with 0.2% NaCl to give xanthan concentrations in the range 0.005–0.03% w/w. The intrinsic viscosity was obtained from a combined Huggins and Kramer extrapolation of the zero shear rate viscosity.

#### 3.2.3 Steady shear and complex viscosity

The steady shear and complex viscosity of the xanthan and starch suspensions or solutions was measured in the rheometer at 20 °C. In this case the rheometer was equipped with sand blasted parallel plate geometry with a diameter of 50 mm and a gap size of 1 mm. Complex viscosity measurements were made in the linear viscoelastic region. This same protocol was also used to measure the rheological properties of the model soups used for sensory testing.

### 3.3 Preparation of model soups for sensory evaluation

The particulate form of xanthan produced by extrusion is believed to be maintained by rearrangement of the dihelical linkages that maintain the native structure. On heating the particulate form above the order disorder transition temperature these linkages are disrupted and on cooling the structure revert to the conventional ordered molecular form (Sereno et al., 2007). Soups were prepared and thickened by starch and the two forms of xanthan. Thickeners were made from extruded xanthan using different heating procedures. Concentrated solutions of molecular xanthan (MX) were created by heating a 2% suspension of extruded xanthan to 95 °C on a hot plate. A viscous concentrated suspension of the particulate form of xanthan (PX) was prepared by heating 3.5% of the extruded material to 65 °C in a water bath. A 7.5% suspension of modified starch (MS) was prepared by heating to 95 °C in the same way as used to prepare MX. All samples contained 0.2% NaCl. Model soups were made by adding tomato juice, water, salt and the concentrated thickener solutions to make soups with 0.63% MX, 0.9% PX and 2% MS. To ensure microbial safety soups were heated to 75 °C in a water bath and cooled to room temperature for presentation to the panellists.

The formulation of the three soups is shown in Table 1.

### 3.4 Sensory evaluation

The three samples were compared for thickness, flavour intensity and saltiness using a multiple pair wise comparison. Experienced panellists (16) from the University of Nottingham external sensory panel were used following pre-screening for sensitivity to salt. The sample size in each sample pot was 15 ml, but no panellist ate all of the sample, most consumed 5–10 ml. The samples were served at room temperature (19 °C). For each test the panellist had to take a 5 ml spoonful of each sample, place in their mouth on their tongue, remove the spoon, allow the sample to coat the roof of their mouth, hold in the mouth for a minimum of 5 s before swallowing, then cleanse their palate with water before tasting the next sample. Significance of the difference was determined using a Friedman Analysis of Rank.

## 4 Results and discussion

Table 2 displays the processing conditions and some properties of the extruded and unprocessed xanthans. A noteworthy feature of the process is the high mechanical energy and this may be responsible for the structural rearrangement which results in the novel particulate properties. This is currently subject to a separate investigation. The milled extrudate showed excellent dispersibility in water and this was similar to the samples prepared using a different extruder and reported in the earlier work by Sereno et al. (2007).

**Fig. 2:**
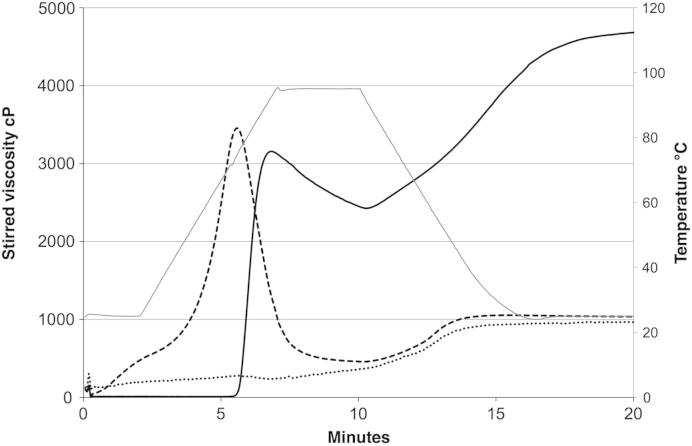
Rapid viscosity analysis response for thickeners ^…^ 2% Unprocessed xanthan, - - - - 2% Extruded xanthan, ^___^ 8% Modified starch all in 0.2% NaCl.

Despite the severity of the extrusion process and subsequent heating and shearing in the Rapid Viscosity Analyser (RVA), the intrinsic viscosity of the extruded xanthan was not significantly different to the unprocessed control suggesting that no degradation occurred. An informative way of characterising the processed material is to obtain a pasting curve using the RVA, as is frequently done for starch and cereal flours. This involved heating with stirring up to 95 °C and subsequent cooling while the viscosity is continuously measured. Fig. 2 shows the viscosity response for the extruded xanthan, the unprocessed xanthan and the modified starch.

The most marked feature of the pasting curve is the rise in viscosity to a peak for the particulate xanthan in a somewhat similar way to that observed for starch. Unprocessed xanthan does not show this type of behaviour. In previous work it was shown that the peak viscosity corresponds well to the temperature of the melting endotherm for xanthan which also increases with salt concentration (Sereno et al., 2007). At the end of the RVA temperature cycle it is believed that the solution structure is that of a conventional molecular solution, whereas prior to this peak temperature the material is in the form of a particulate suspension. The increase in viscosity for the processed form of xanthan with temperature is believed to be due to particle swelling in a similar way to that observed with starch. If the heating process is stopped before the viscosity peaks the system will behave as a suspension of swollen particles. At the end of the heating process in the RVA, conversion to the conventional molecular form will have been achieved. This is supported by the similar viscosities between the unprocessed control and the processed xanthan observed at the end of the RVA heating process.

**Fig. 3:**
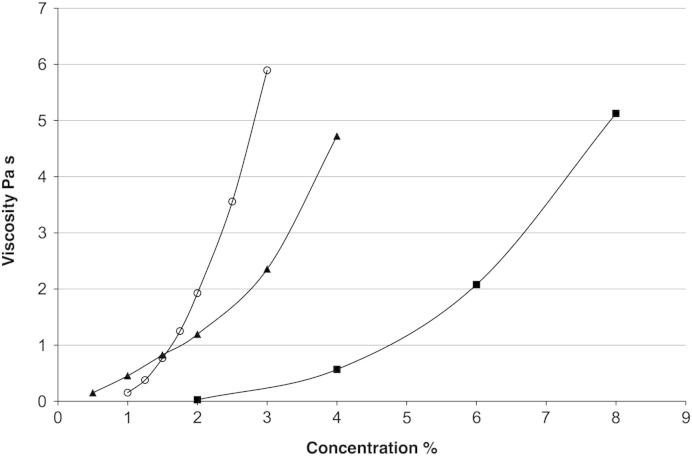
Concentration dependence of viscosity measured at 50 s^−1^ for three different thickening systems. ▴ Molecular xanthan (MX), ○ particulate xanthan (PX), ■ modified starch (MS).

Fig. 3 compares the steady shear viscosities, measured at a shear rate of 50 s^−1^, of the two forms of xanthan and starch as a function of concentration in 0.2% NaCl.

The particulate form was prepared by stopping the RVA when the temperature during the heating cycle had reached 75 °C. The molecular form and the starch were also prepared in the RVA prior to measurement in the rheometer. In these cases the samples were taken at the end of the full heating cycle. The data are presented at a shear rate of 50 s^−1^, since this condition is frequently regarded as a shear rate appropriate to that found in the mouth, although more complex relationships have been proposed (Stokes, 2012).

The concentration dependence of the viscosity shown in Fig. 3 is very different between the two forms of the xanthan. The curves cross at a concentration of about 1%; the particulate form showing much poorer thickening than the molecular form at low concentrations, but higher at higher concentrations. This behaviour would be expected when the viscosity of suspensions is compared with polymer solutions.

**Fig. 4:**
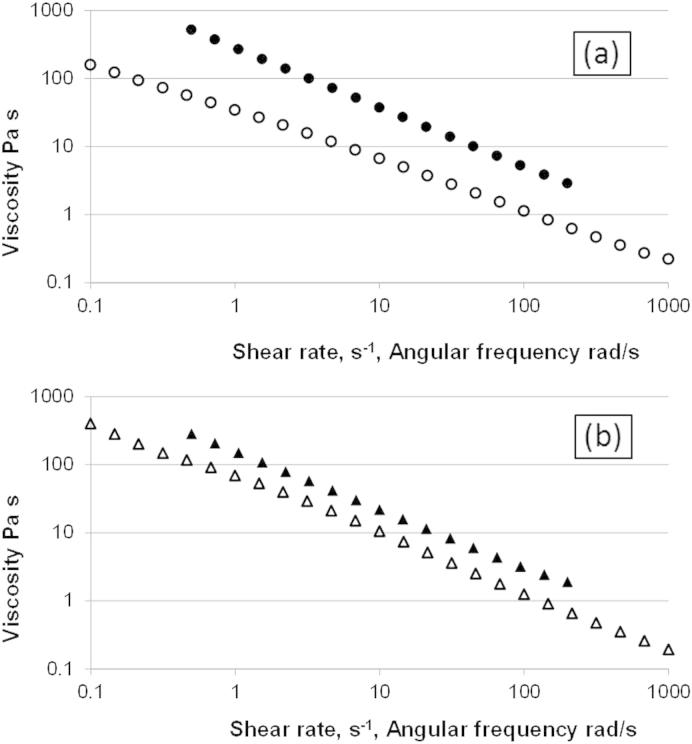
Dependence of shear viscosity (open symbols) and complex viscosity (filled symbols) on respectively shear rate and angular frequency. (a) 2% Extruded particulate xanthan (PX), (b) 3% Extruded molecular xanthan (MX).

When the frequency dependence and shear rate dependence of viscosity are compared neither of the xanthan samples obey the Cox Merz rule (Fig. 4). This departure from Cox Merz is frequently observed for hydrocolloid solutions including xanthan (Lee & Brant, 2002).

### 4.1 Mixing

**Fig. 5:**
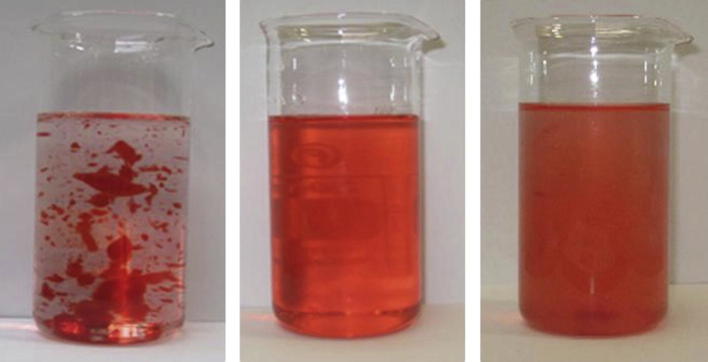
Comparison of the mixing behaviour of 1.25% extruded molecular xanthan (MX) (left) 1.5% extruded particulate xanthan (PX) (middle) and 4.5% modified starch (MS) (right) with 0.2% NaCl, in water.

As mentioned in the introduction it has been postulated that particulate thickened structures mix well with water in contrast to molecularly thickened structures above c*. The comparison between the three systems of interest, namely starch and the two forms of xanthan, are shown in Fig. 5. In this case a small amount of red dye has been added to systems and 5 g of this coloured solution mixed with 200 ml of water by gently stirring by hand (three times with a spatula). The samples were photographed after about 10 s after the stirring had ceased. The differences between the three systems are clear, with the particulate form mixing much better than the molecular form.

### 4.2 Properties of soups used for sensory perception

The results of the mixing experiments suggest that the product thickened by particulate xanthan would have sensory properties closer to starch thickened products than products thickened by molecular xanthan. To test this in a system closer to a real product three model tomato soups were prepared thickened by the two forms of xanthan and starch. Salt was added to give a total concentration of 0.2%. The formulations of the three products tasted have been previously given in Table 1.

Table 3 shows the measured viscosities under steady shear and dynamic conditions. For our experiments we matched the products, as is conventionally done on the basis of equal steady shear viscosities. However, it has been suggested that this underestimates the perceived thickness of xanthan thickened products. A better prediction may be obtained using a complex viscosity measured at 100 rad/s and therefore these values have been included in Table 3 (Richardson, Morris, Ross-Murphy, Taylor & Dea, 1989). The samples were mixed in water, as described for the samples associated with Fig. 5, except in this case it was not necessary to add a red dye because the soup was already coloured. The soups thickened with the three systems showed mixing behaviour that was very similar to that shown in Fig. 5 for the xanthan and starch suspensions/solutions. This is to say soups thickened with conventional molecular xanthan mixed poorly, whereas starches thickened with starch and particulate xanthan mixed well.

### 4.3 Sensory perception

**Fig. 6:**
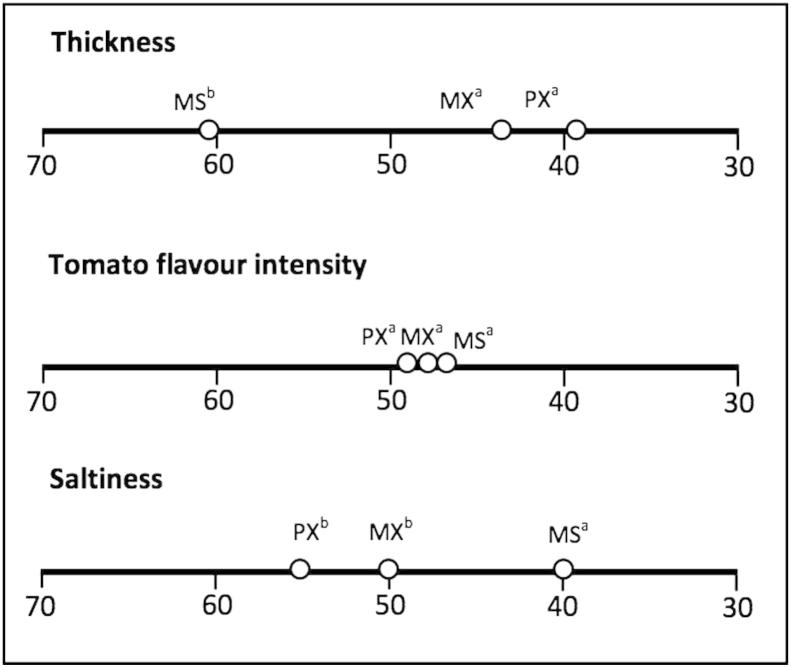
Rank sum scores for each sample for thickness, tomato flavour intensity and saltiness. Samples identified with the same superscript letter codes are not significantly different. Decreasing numerical values correspond to an increase in the attribute. Extruded molecular xanthan (MX), Extruded particulate xanthan (PX) and modified starch (MS).

Fig. 6 summarises the results from the sensory panel.

Despite the high level of discrimination expected from an experienced panel in a paired comparison test no differences were found between the tomato flavour intensity of the three products. Using a magnitude estimation approach we have previously found that starch, if it maintains its granular structure, does not reduce flavour perception at high viscosity (Ferry et al., 2006). Morris (1993) in a study on xanthan thickened systems, also using magnitude estimation, reported that there was no reduction in perceived flavour or sweetness at xanthan concentrations as high as 1%. Our results are in agreement with this with respect to flavour perception. Also consistent with previous work is the higher perceived thickness for xanthan. The complex viscosity at 100 rad/s (Table 3) would seem to be a better predictor of the perceived thickness. The reasons for this are unclear. One possibility is that because of its weak gel structure xanthan shows stress overshoot on deformation and this additional stress component is not taken into account in shear viscosity measurements.

Our results show no difference between the two forms of xanthan for any of the attributes. There was a lower perceived saltiness for both forms of xanthan compared with starch. The lack of difference between the two forms of xanthan, which mix very differently with water, is not consistent with our previous hypothesis. This hypothesis was successful in explaining: the reduction in taste and flavour perception for other hydrocolloids once the concentration exceeded c*, the differences in perception between starch and hydrocolloid thickened solutions, and the relationship between starch structure and perception. From earlier studies it is clear that xanthan behaves differently to other hydrocolloids. In particular it has been shown that when xanthan is mixed with a wide range of polyelectrolytes (alginate, carboxymethyl cellulose, low methoxyl pectin, λ-carrageenen and sodium polyacrylate) conversion to a strand-like particulate structure can occur (Boyd et al., 2009). This behaviour appears to be unique to xanthan. Saliva contains two forms of mucins that are responsible for the lubricating and viscosifying properties of saliva (Tabak, 1995). These glycoproteins are polyelectrolyes and it is possible that they can interact with xanthan in a similar way to the other polyelectrolyes investigated. This is very speculative at this stage, but if such an interaction did occur in the mouth with the dramatic change in rheology reported by Boyd et al. (2009) it could not only explain the excellent taste perception of xanthan demonstrated here, but could be the basis of new biomedical applications for xanthan gum.

## 5 Conclusions

The link between the mixing behaviour in water of viscous solutions of starch and many hydrocolloids with flavour and taste perception does not hold for xanthan gum. The xanthan can show poor mixing, but comparable flavour perception with a modified starch thickened system that mixes well. The rheology in the mouth may be different for the xanthans. Conversion of xanthan to the particulate form by extrusion processing does not appear to confer any sensory advantage, however it should be noted that the complex viscosity of this material was much higher than the starch when assessed at 100 rad/s. The particulate form of xanthan showed ease of dispersability and mixing. The molecular xanthan material did not show differences in taste and flavour perception, when compared to starch and these materials had similar viscosities at 100 rad/s. Starch and xanthan may be atypical for hydrocolloids where increasing viscosity is expected to lower sensory quality. A hypothesis that could explain the results for the xanthans is that the xanthan may interact with mucins in a similar way to that reported for a range of negatively charged polyelectrolytes, but this has yet to be tested.

## Figures and Tables

**Table 1:** Formulation of model tomato soups thickened with extruded molecular xanthan (MX), extruded particulate xanthan (PX) and modified starch (MS).

Ingredient %	Xanthan		Modified starch (MS)
	Molecular (MX)	Particulate (PX)	
Thickener	0.6	0.9	2.0
Salt	0.2	0.2	0.2
Water	32.7	32.4	31.3
Tomato juice	66.5	66.5	66.5

**Table 2:** Process conditions and product characteristics of the extruded xanthan.

Process parameters	Values
Moisture content exit die (% wwb)	40.8
Specific mechanical energy (kJ/kg)	1580
Product temperature exit die (°C)	95–98
Intrinsic viscosity extruded xanthan (dl/g)	50.8
Intrinsic viscosity of non processed control (dl/g)	50.5
Residence time (s)	51.0
Moisture content of final dried product (% wwb)	10.7
Moisture content of unprocessed control (% wwb)	12.0
Output exit die (kg/h)	1.9

**Table 3:** Comparison of shear and complex viscosity values for model tomato soup.

Thickener	Steady shear viscosity at 50 s^−1^ (Pa s)	Power law index^a^	Complex viscosity at 100 rad/s (Pa s)
Molecular xanthan (MX) (0.6%)	0.457	0.18	0.8
Particulate xanthan (PX) (0.9%)	0.454	0.22	1.67
Modified starch (MS) (2%)	0.495	0.23	0.63

a The power law index(s) was obtained by fitting the steady shear viscosity *η* to the relationship *η* = *K*${\dot{\gamma }}^{s-1}$, where *K* is a constant.
